# 2-Methyl­sulfanyl-9*H*-1,3,4-thia­diazolo[2,3-*b*]quinazolin-9-one

**DOI:** 10.1107/S1600536812026189

**Published:** 2012-06-20

**Authors:** Adel S. El-Azab, Alaa A.-M. Abdel-Aziz, Ibrahim A. Al-Swaidan, Seik Weng Ng, Edward R. T. Tiekink

**Affiliations:** a Department of Pharmaceutical Chemistry, College of Pharmacy, King Saud University, Riyadh 11451, Saudi Arabia; b Department of Organic Chemistry, Faculty of Pharmacy, Al-Azhar University, Cairo 11884, Egypt; c Department of Medicinal Chemistry, Faculty of Pharmacy, University of Mansoura, Mansoura 35516, Egypt; d Department of Chemistry, University of Malaya, 50603 Kuala Lumpur, Malaysia; e Chemistry Department, Faculty of Science, King Abdulaziz University, PO Box 80203 Jeddah, Saudi Arabia

## Abstract

In the title compound, C_10_H_7_N_3_OS_2_, the 16 non-H atoms are almost planar (r.m.s. deviation = 0.037 Å) and the S-bound methyl group is *syn* to the ketone O atom. In the crystal, centrosymmetrically related mol­ecules are connected by pairs of C—H⋯O inter­actions between the ketone O and methyl H atoms. The dimeric aggregates are connected into a linear supra­molecular chain along the *b* axis *via* π–π inter­actions occurring between the five-membered and benzene rings [centroid–centroid distance = 3.6123 (9) Å]. The chains assemble into layers in the *bc* plane *via* S⋯S inter­actions involving the endocyclic S atoms [S⋯S = 3.4607 (6) and 3.4792 (6) Å].

## Related literature

For recent studies on the synthesis and biological properties of quinazoline-4(3*H*)-one derivatives, see: El-Azab & ElTahir (2012 ▶); El-Azab *et al.* (2011 ▶). For the synthesis and anti-microbial activity of the title compound, see: El-Azab (2007 ▶).
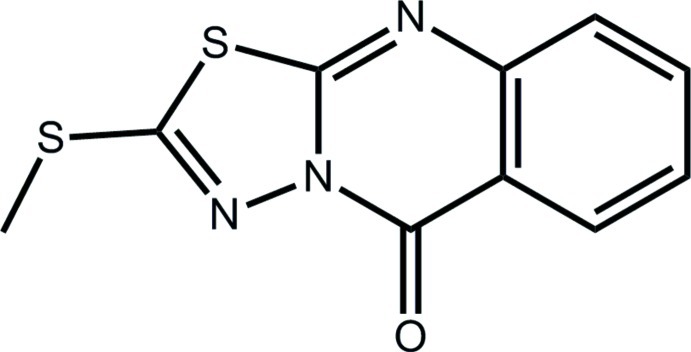


## Experimental

### 

#### Crystal data

C_10_H_7_N_3_OS_2_
*M* *_r_* = 249.31
Monoclinic, 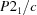

*a* = 11.8193 (4) Å
*b* = 4.9841 (2) Å
*c* = 17.4985 (6) Å
β = 91.453 (3)°
*V* = 1030.48 (6) Å^3^
*Z* = 4
Cu *K*α radiation
μ = 4.53 mm^−1^
*T* = 100 K
0.30 × 0.10 × 0.03 mm

#### Data collection

Agilent SuperNova Dual diffractometer with an Atlas detector
Absorption correction: multi-scan (*CrysAlis PRO*; Agilent, 2012 ▶) *T*_min_ = 0.344, *T*_max_ = 0.876
3781 measured reflections
2110 independent reflections
1937 reflections with *I* > 2σ(*I*)
*R*_int_ = 0.018

#### Refinement

*R*[*F* ^2^ > 2σ(*F* ^2^)] = 0.029
*wR*(*F* ^2^) = 0.082
*S* = 1.09
2110 reflections
145 parameters
H-atom parameters constrained
Δρ_max_ = 0.36 e Å^−3^
Δρ_min_ = −0.27 e Å^−3^

### 

Data collection: *CrysAlis PRO* (Agilent, 2012 ▶); cell refinement: *CrysAlis PRO*; data reduction: *CrysAlis PRO*; program(s) used to solve structure: *SHELXS97* (Sheldrick, 2008 ▶); program(s) used to refine structure: *SHELXL97* (Sheldrick, 2008 ▶); molecular graphics: *ORTEP-3* (Farrugia, 1997 ▶) and *DIAMOND* (Brandenburg, 2006 ▶); software used to prepare material for publication: *publCIF* (Westrip, 2010 ▶).

## Supplementary Material

Crystal structure: contains datablock(s) global, I. DOI: 10.1107/S1600536812026189/hb6843sup1.cif

Structure factors: contains datablock(s) I. DOI: 10.1107/S1600536812026189/hb6843Isup2.hkl

Supplementary material file. DOI: 10.1107/S1600536812026189/hb6843Isup3.cml

Additional supplementary materials:  crystallographic information; 3D view; checkCIF report

## Figures and Tables

**Table 1:** Hydrogen-bond geometry (Å, °)

*D*—H⋯*A*	*D*—H	H⋯*A*	*D*⋯*A*	*D*—H⋯*A*
C10—H10*A*⋯O1^i^	0.98	2.32	3.170 (2)	145

Symmetry code: (i) 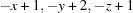.

**Fig. 1.**
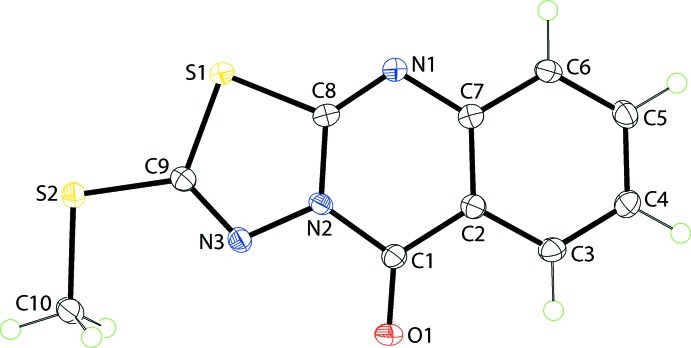
The molecular structure of (I) showing displacement ellipsoids at the 50% probability level.

**Fig. 2.**
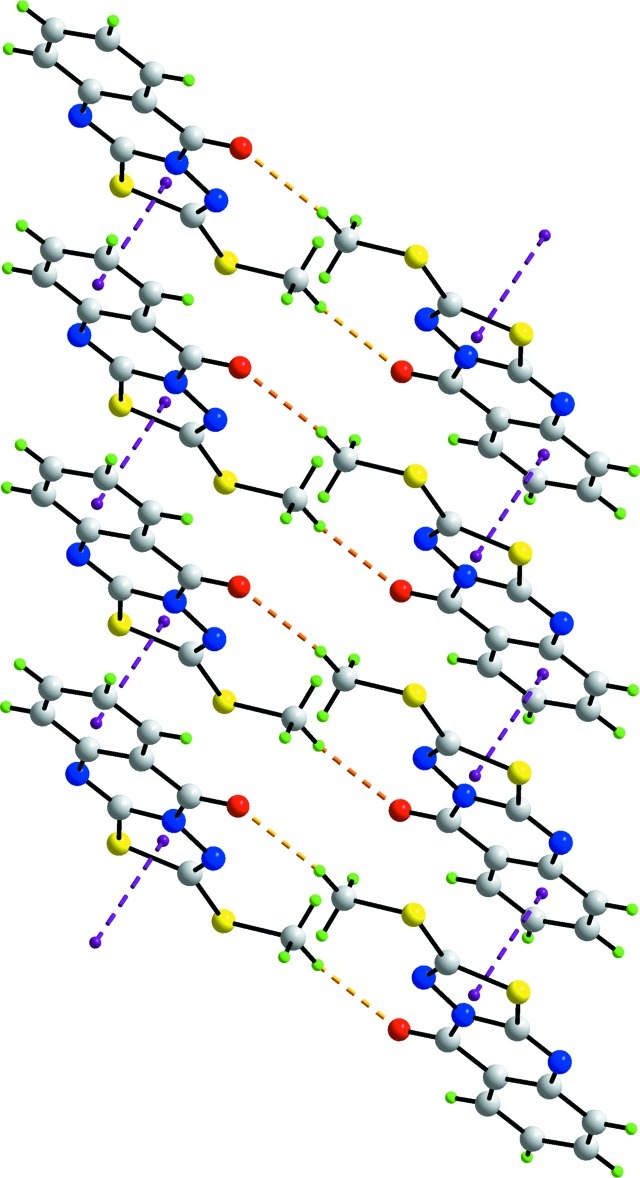
A view of the linear supramolecular chain along the b axis in (I). The C—H···O and π—π interactions are shown as orange and purple dashed lines respectively.

**Fig. 3.**
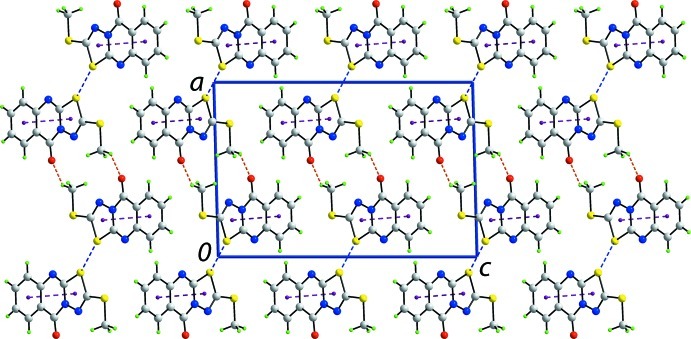
A view in projection down the b axis of the unit-cell contents for (I). The C—H···O, π—π and S···S interactions are shown as orange, purple and blue dashed lines respectively.

## supplementary crystallographic information

### Comment

Quinazoline-4(3*H*)-one derivatives attract interest owing to their putative biological activity (El-Azab & ElTahir, 2012; El-Azab *et al.*, 2011). The title compound, 2-(methylthio)-5*H*-[1,3,4]thiadiazolo[2,3-*b*]quinazolin-5-one (I), has been synthesized previously and evaluated for its anti-microbial activity (El-Azab, 2007). Herein, we describe its crystal structure determination.

The 16 non-hydrogen atoms in (I), Fig. 1, are planar with the r.m.s. deviation being 0.037 Å. The maximum deviations from the least-squares plane are 0.068 (1) Å for the ketone-O1 atom and -0.065 (2) Å for the methyl-C10 atom. The *S*-bound methyl group is *syn* to the ketone-O1 atom.

In the crystal packing, centrosymmetrically related molecules are connected by C—H···O interactions between the ketone-O and methyl-H atoms, Table 1, *via* a 16-membered {···HCSCN_2_CO}_2_ synthon, Fig. 2. The dimeric aggregates are connected into a linear supramolecular chain along the *b* axis *via*π—π interactions occurring between the five-membered and benzene rings [inter-centroid distance = 3.6123 (9) Å, angle of inclination = 2.09 (7)° for symmetry operation: *x*, 1 + *y*, *z*]. The chains assemble into layers in the *bc* plane *via* S···S interactions involving the endocyclic-S1 atoms whereby each S1 atom forms two such interactions [S1···S1^i^ = 3.4607 (6) Å for symmetry operation *i*: 2 - *x*,2 - *y*,1 - *z*; and S1···S1^ii^ = 3.4792 (6) Å for *ii*: 2 - *x*, 1 - *y*, 1 - *z*]. Layers stack along the *a* axis without specific interactions between them, Fig. 3.

### Experimental

A mixture of 2-mercapto-5*H*-[1,3,4]thiadiazolo[2,3-*b*]quinazolin-5-one (470 mg, 2 mmol) and methyliodide (2.1 mmol) in acetone (10 ml) containing anhydrous potassium carbonate (300 mg) was stirred at room temperature for 12 h. The reaction mixture was filtered, the solvent removed under reduced pressure and the solid obtained was dried and recrystallized from ethanol. Yield 88%. ^1^H NMR (CDCl_3_): δ 8.42 (d, 1H, J = 7.5 Hz), 7.79 (t, 1H, J = 7.0 Hz), 7.63 (d, 1H, J = 8.0 Hz), 7.49 (t, 1H, J = 7.0 Hz), 2.84 (s, 3H) p.p.m.. ^13^C NMR (CDCl_3_): δ = 15.3, 118.9, 126.2, 127.6, 134.8, 147.2, 156.2, 157.1, 158.5 p.p.m..

### Refinement

Carbon-bound H-atoms were placed in calculated positions [C—H = 0.95 to 0.98 Å, *U*_iso_(H) = 1.2–1.5*U*_eq_(C)] and were included in the refinement in the riding model approximation.

### Crystal data

**Table:**

C_10_H_7_N_3_OS_2_	*F*(000) = 512
*M**_r_* = 249.31	*D*_x_ = 1.607 Mg m^−^^3^
Monoclinic, *P*2_1_/*c*	Cu *K*α radiation, λ = 1.54184 Å
Hall symbol: -P 2ybc	Cell parameters from 2166 reflections
*a* = 11.8193 (4) Å	θ = 3.7–76.5°
*b* = 4.9841 (2) Å	µ = 4.53 mm^−^^1^
*c* = 17.4985 (6) Å	*T* = 100 K
β = 91.453 (3)°	Prism, colourless
*V* = 1030.48 (6) Å^3^	0.30 × 0.10 × 0.03 mm
*Z* = 4	

### Data collection

**Table:**

Agilent SuperNova Dual diffractometer with an Atlas detector	2110 independent reflections
Radiation source: SuperNova (Cu) X-ray Source	1937 reflections with *I* > 2σ(*I*)
Mirror monochromator	*R*_int_ = 0.018
Detector resolution: 10.4041 pixels mm^-1^	θ_max_ = 76.7°, θ_min_ = 3.7°
ω scan	*h* = −12→14
Absorption correction: multi-scan (*CrysAlis PRO*; Agilent, 2012)	*k* = −5→6
*T*_min_ = 0.344, *T*_max_ = 0.876	*l* = −15→21
3781 measured reflections	

### Refinement

**Table:**

Refinement on *F*^2^	Primary atom site location: structure-invariant direct methods
Least-squares matrix: full	Secondary atom site location: difference Fourier map
*R*[*F*^2^ > 2σ(*F*^2^)] = 0.029	Hydrogen site location: inferred from neighbouring sites
*wR*(*F*^2^) = 0.082	H-atom parameters constrained
*S* = 1.09	*w* = 1/[σ^2^(*F*_o_^2^) + (0.0457*P*)^2^ + 0.3686*P*] where *P* = (*F*_o_^2^ + 2*F*_c_^2^)/3
2110 reflections	(Δ/σ)_max_ = 0.001
145 parameters	Δρ_max_ = 0.36 e Å^−^^3^
0 restraints	Δρ_min_ = −0.27 e Å^−^^3^

### Fractional atomic coordinates and isotropic or equivalent isotropic displacement parameters (Å^2^)

**Table:**

	*x*	*y*	*z*	*U*_iso_*/*U*_eq_	
S1	0.90968 (3)	0.75129 (8)	0.46619 (2)	0.01702 (12)	
S2	0.76245 (3)	1.12015 (8)	0.55613 (2)	0.01660 (12)	
N1	0.90012 (11)	0.3645 (3)	0.35873 (8)	0.0160 (3)	
N2	0.72981 (11)	0.5607 (3)	0.40394 (7)	0.0145 (3)	
N3	0.68915 (11)	0.7516 (3)	0.45394 (7)	0.0160 (3)	
O1	0.55197 (9)	0.4218 (3)	0.36566 (7)	0.0217 (3)	
C1	0.65417 (13)	0.4007 (3)	0.36022 (9)	0.0159 (3)	
C2	0.71312 (13)	0.2152 (3)	0.31034 (9)	0.0153 (3)	
C3	0.64945 (13)	0.0467 (4)	0.26154 (9)	0.0183 (3)	
H3	0.5691	0.0533	0.2616	0.022*	
C4	0.70332 (14)	−0.1282 (4)	0.21351 (9)	0.0196 (3)	
H4	0.6602	−0.2429	0.1806	0.024*	
C5	0.82196 (14)	−0.1368 (3)	0.21325 (9)	0.0192 (3)	
H5	0.8589	−0.2560	0.1796	0.023*	
C6	0.88539 (13)	0.0261 (3)	0.26135 (9)	0.0179 (3)	
H6	0.9657	0.0173	0.2609	0.021*	
C7	0.83228 (13)	0.2050 (3)	0.31091 (9)	0.0149 (3)	
C8	0.84595 (12)	0.5289 (3)	0.40102 (8)	0.0149 (3)	
C9	0.77423 (13)	0.8644 (3)	0.48930 (9)	0.0152 (3)	
C10	0.61018 (14)	1.1556 (4)	0.55743 (10)	0.0211 (3)	
H10A	0.5903	1.2965	0.5938	0.032*	
H10B	0.5762	0.9854	0.5730	0.032*	
H10C	0.5815	1.2041	0.5062	0.032*	

### Atomic displacement parameters (Å^2^)

**Table:**

	*U*^11^	*U*^22^	*U*^33^	*U*^12^	*U*^13^	*U*^23^
S1	0.01263 (19)	0.0180 (2)	0.0204 (2)	−0.00013 (13)	−0.00028 (14)	−0.00389 (14)
S2	0.0170 (2)	0.0176 (2)	0.0151 (2)	0.00105 (14)	−0.00022 (14)	−0.00248 (14)
N1	0.0136 (6)	0.0176 (7)	0.0167 (6)	0.0001 (5)	0.0003 (5)	−0.0019 (5)
N2	0.0124 (6)	0.0179 (7)	0.0133 (6)	0.0031 (5)	0.0007 (5)	−0.0011 (5)
N3	0.0161 (6)	0.0180 (7)	0.0139 (6)	0.0031 (5)	0.0001 (5)	−0.0018 (5)
O1	0.0118 (5)	0.0294 (7)	0.0239 (6)	0.0024 (5)	−0.0007 (4)	−0.0067 (5)
C1	0.0148 (7)	0.0191 (7)	0.0138 (7)	0.0014 (6)	−0.0011 (6)	−0.0006 (6)
C2	0.0151 (7)	0.0176 (7)	0.0131 (7)	0.0011 (6)	−0.0001 (5)	0.0006 (6)
C3	0.0146 (7)	0.0227 (8)	0.0176 (7)	0.0013 (7)	−0.0018 (6)	−0.0009 (6)
C4	0.0190 (8)	0.0219 (8)	0.0177 (8)	0.0007 (6)	−0.0041 (6)	−0.0031 (6)
C5	0.0204 (8)	0.0207 (8)	0.0166 (7)	0.0037 (6)	0.0012 (6)	−0.0032 (6)
C6	0.0135 (7)	0.0206 (8)	0.0195 (7)	0.0022 (6)	0.0013 (6)	−0.0013 (6)
C7	0.0149 (7)	0.0160 (7)	0.0139 (7)	−0.0004 (6)	0.0001 (5)	0.0017 (6)
C8	0.0127 (7)	0.0159 (7)	0.0160 (7)	−0.0008 (6)	−0.0005 (5)	0.0023 (6)
C9	0.0155 (7)	0.0160 (7)	0.0141 (7)	0.0019 (6)	0.0005 (5)	0.0014 (6)
C10	0.0178 (7)	0.0261 (9)	0.0194 (8)	0.0048 (7)	0.0015 (6)	−0.0032 (7)

### Geometric parameters (Å, º)

**Table:**

S1—C8	1.7473 (16)	C2—C7	1.409 (2)
S1—C9	1.7542 (16)	C3—C4	1.378 (2)
S2—C9	1.7378 (16)	C3—H3	0.9500
S2—C10	1.8091 (17)	C4—C5	1.403 (2)
N1—C8	1.286 (2)	C4—H4	0.9500
N1—C7	1.393 (2)	C5—C6	1.378 (2)
N2—C8	1.3840 (18)	C5—H5	0.9500
N2—N3	1.3866 (18)	C6—C7	1.403 (2)
N2—C1	1.409 (2)	C6—H6	0.9500
N3—C9	1.296 (2)	C10—H10A	0.9800
O1—C1	1.2186 (19)	C10—H10B	0.9800
C1—C2	1.461 (2)	C10—H10C	0.9800
C2—C3	1.403 (2)		
			
C8—S1—C9	88.48 (7)	C6—C5—H5	119.7
C9—S2—C10	100.19 (8)	C4—C5—H5	119.7
C8—N1—C7	114.96 (13)	C5—C6—C7	120.47 (14)
C8—N2—N3	117.51 (13)	C5—C6—H6	119.8
C8—N2—C1	122.09 (13)	C7—C6—H6	119.8
N3—N2—C1	120.37 (12)	N1—C7—C6	118.30 (14)
C9—N3—N2	108.78 (13)	N1—C7—C2	122.93 (14)
O1—C1—N2	121.69 (14)	C6—C7—C2	118.77 (14)
O1—C1—C2	126.16 (15)	N1—C8—N2	127.10 (14)
N2—C1—C2	112.15 (13)	N1—C8—S1	124.55 (12)
C3—C2—C7	120.23 (14)	N2—C8—S1	108.35 (11)
C3—C2—C1	119.08 (14)	N3—C9—S2	124.42 (12)
C7—C2—C1	120.69 (14)	N3—C9—S1	116.88 (12)
C4—C3—C2	120.06 (15)	S2—C9—S1	118.69 (9)
C4—C3—H3	120.0	S2—C10—H10A	109.5
C2—C3—H3	120.0	S2—C10—H10B	109.5
C3—C4—C5	119.90 (15)	H10A—C10—H10B	109.5
C3—C4—H4	120.0	S2—C10—H10C	109.5
C5—C4—H4	120.0	H10A—C10—H10C	109.5
C6—C5—C4	120.56 (15)	H10B—C10—H10C	109.5
			
C8—N2—N3—C9	−0.24 (19)	C3—C2—C7—N1	−179.16 (15)
C1—N2—N3—C9	177.60 (13)	C1—C2—C7—N1	0.9 (2)
C8—N2—C1—O1	176.66 (15)	C3—C2—C7—C6	0.7 (2)
N3—N2—C1—O1	−1.1 (2)	C1—C2—C7—C6	−179.20 (15)
C8—N2—C1—C2	−3.1 (2)	C7—N1—C8—N2	0.4 (2)
N3—N2—C1—C2	179.13 (13)	C7—N1—C8—S1	179.87 (11)
O1—C1—C2—C3	1.9 (3)	N3—N2—C8—N1	−179.87 (15)
N2—C1—C2—C3	−178.30 (14)	C1—N2—C8—N1	2.3 (2)
O1—C1—C2—C7	−178.17 (16)	N3—N2—C8—S1	0.61 (17)
N2—C1—C2—C7	1.6 (2)	C1—N2—C8—S1	−177.18 (12)
C7—C2—C3—C4	−0.5 (2)	C9—S1—C8—N1	179.88 (15)
C1—C2—C3—C4	179.44 (15)	C9—S1—C8—N2	−0.59 (11)
C2—C3—C4—C5	−0.3 (3)	N2—N3—C9—S2	178.77 (11)
C3—C4—C5—C6	0.8 (3)	N2—N3—C9—S1	−0.27 (17)
C4—C5—C6—C7	−0.5 (3)	C10—S2—C9—N3	0.76 (16)
C8—N1—C7—C6	178.13 (15)	C10—S2—C9—S1	179.79 (10)
C8—N1—C7—C2	−2.0 (2)	C8—S1—C9—N3	0.53 (13)
C5—C6—C7—N1	179.67 (15)	C8—S1—C9—S2	−178.57 (10)
C5—C6—C7—C2	−0.2 (2)		

### Hydrogen-bond geometry (Å, º)

**Table:**

*D*—H···*A*	*D*—H	H···*A*	*D*···*A*	*D*—H···*A*
C10—H10*A*···O1^i^	0.98	2.32	3.170 (2)	145

Symmetry code: (i) −*x*+1, −*y*+2, −*z*+1.
